# Supplementary material for: Nucleosome acidic patch-targeting binuclear ruthenium compounds induce aberrant chromatin condensation
Source: Nat Commun. 2017 Nov 17;8:1575. doi: 10.1038/s41467-017-01680-4 (PMC5691193; doi:10.1038/s41467-017-01680-4)
Supplement: Supplementary file 1 — Supplementary Information [file 41467_2017_1680_MOESM1_ESM.pdf]

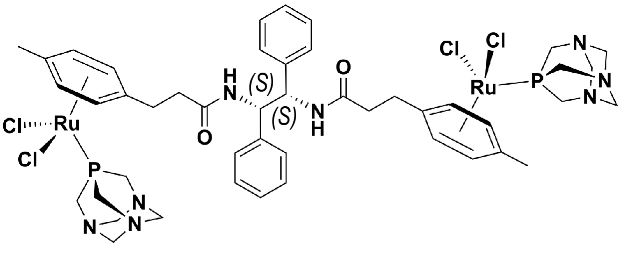

SS

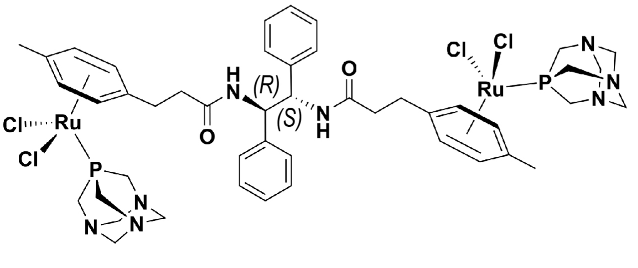

RS

**Supplementary Figure 1.** Structures for additional compounds used in the X-ray crystallographic structural investigations.

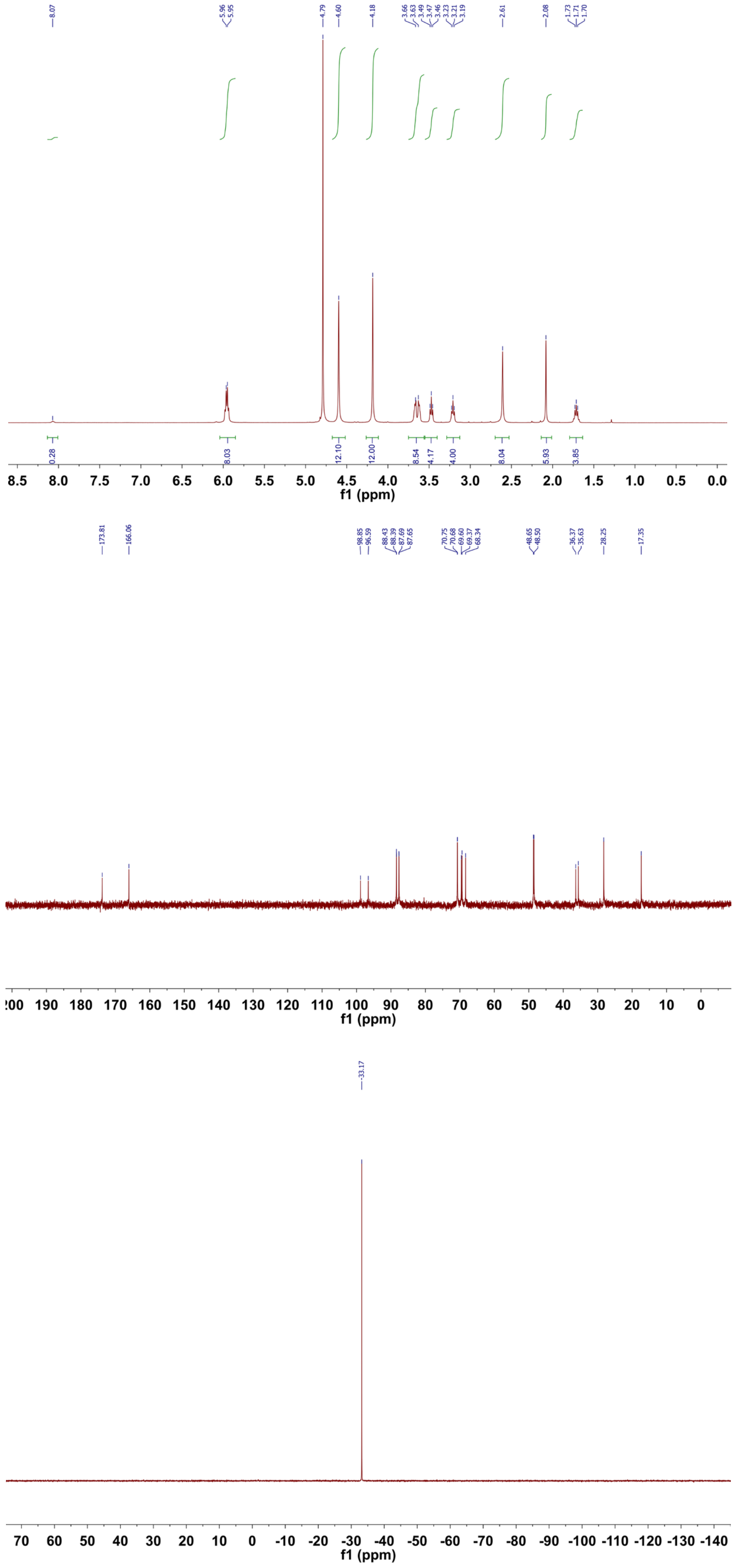


**Supplementary Figure 2.** $^{1}H$ (top), $^{13}C\{^{1}H\}$ (middle) and $^{31}P\{^{1}H\}$ (bottom) NMR spectra ($D_2O$) of RuPEGOx.

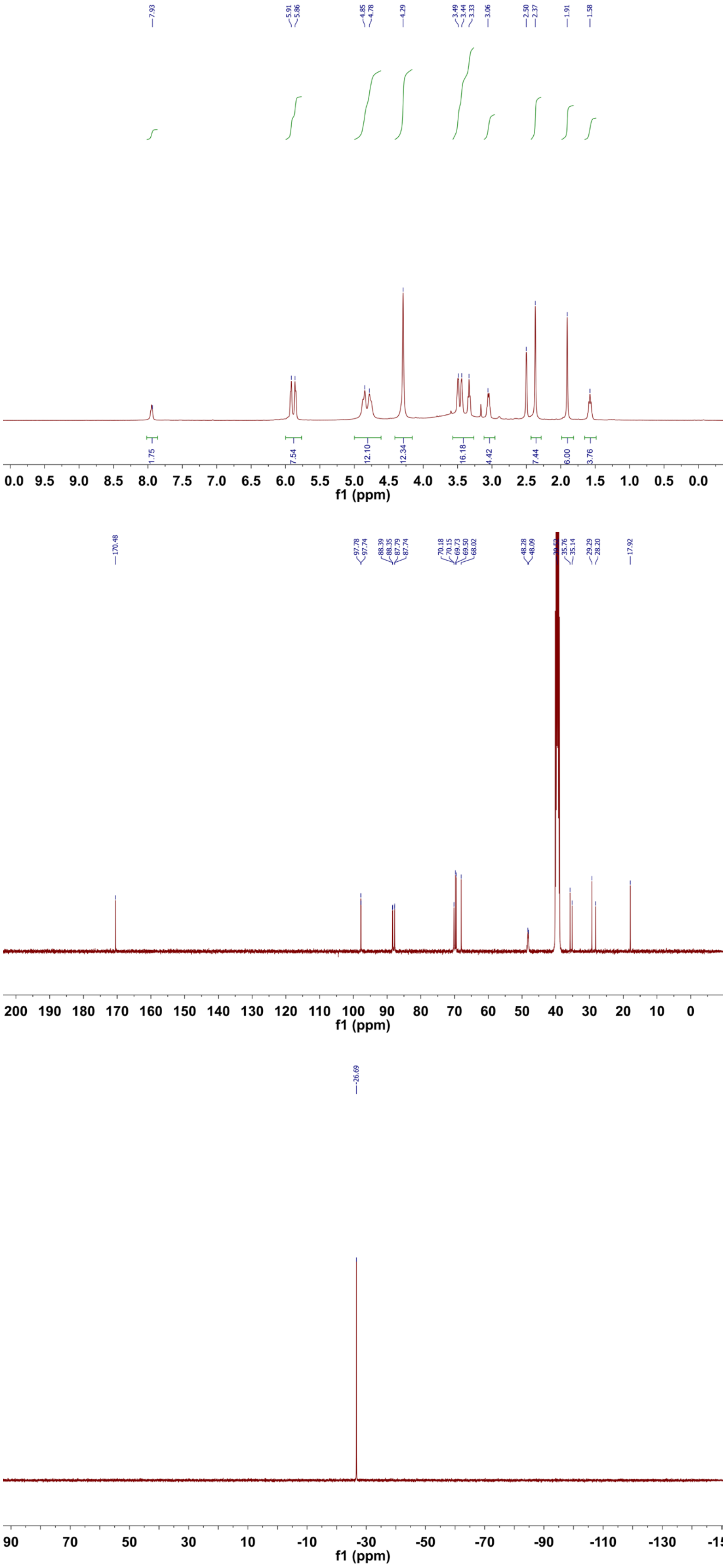


**Supplementary Figure 3.** $^{1}H$ (top), $^{13}C\{^{1}H\}$ (middle) and $^{31}P\{^{1}H\}$ (bottom) NMR spectra (DMSO-*d*6) of RuPEGCl4.

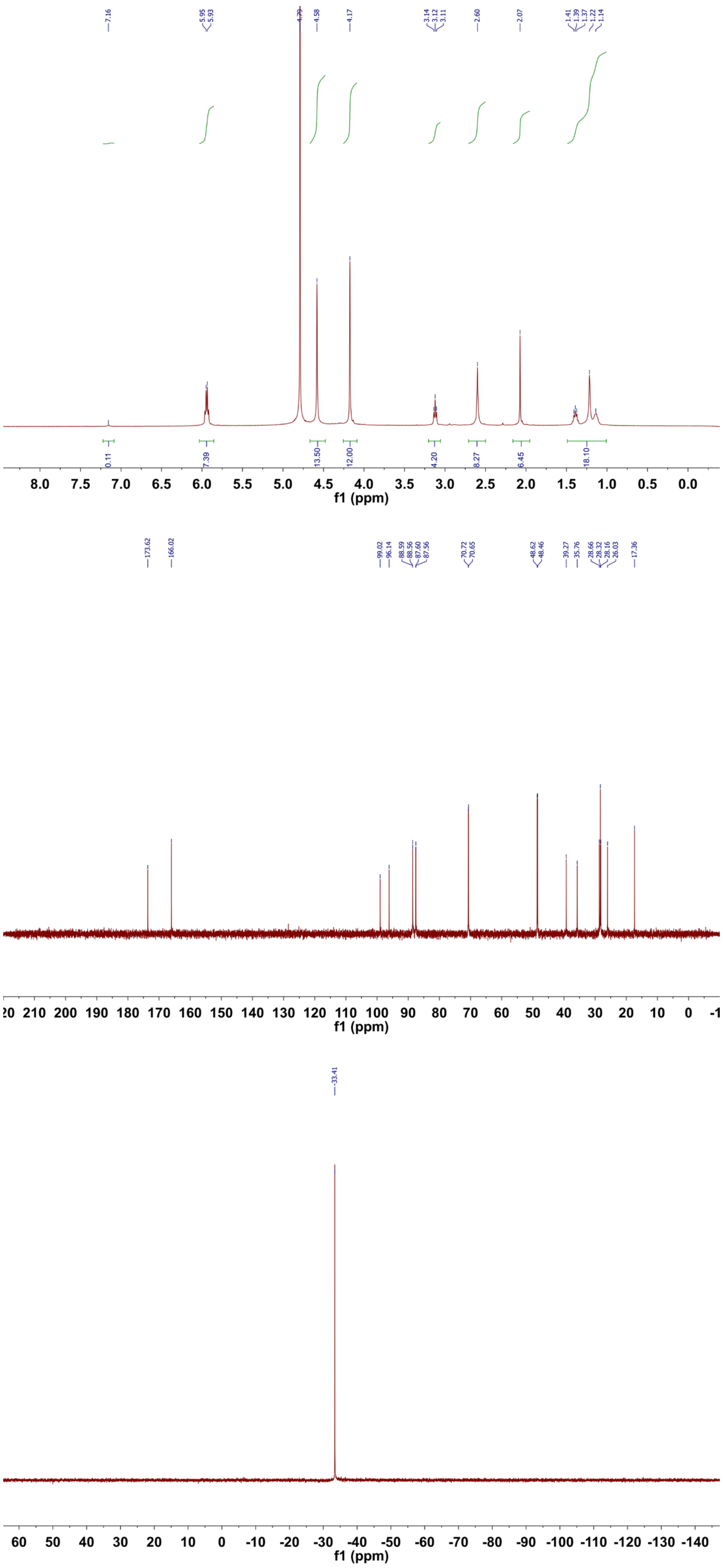

**Supplementary Figure 4.** $^{1}H$ (top), $^{13}C\{^{1}H\}$ (middle) and $^{31}P\{^{1}H\}$ (bottom) NMR spectra ($D_2O$) of RuC10Ox.

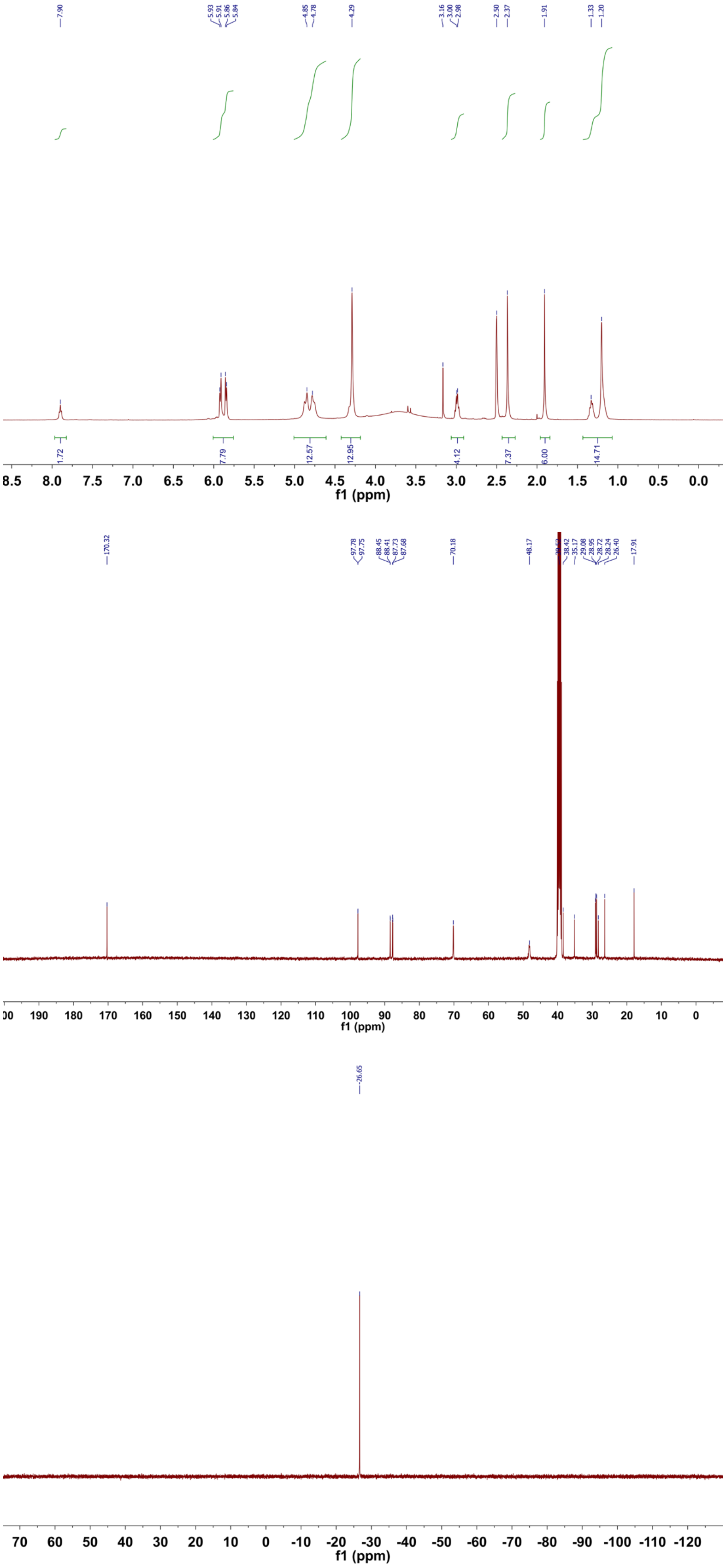


**Supplementary Figure 5.** $^{1}H$ (top), $^{13}C\{^{1}H\}$ (middle) and $^{31}P\{^{1}H\}$ (bottom) NMR spectra (DMSO-*d*6) of RuC10Cl4.

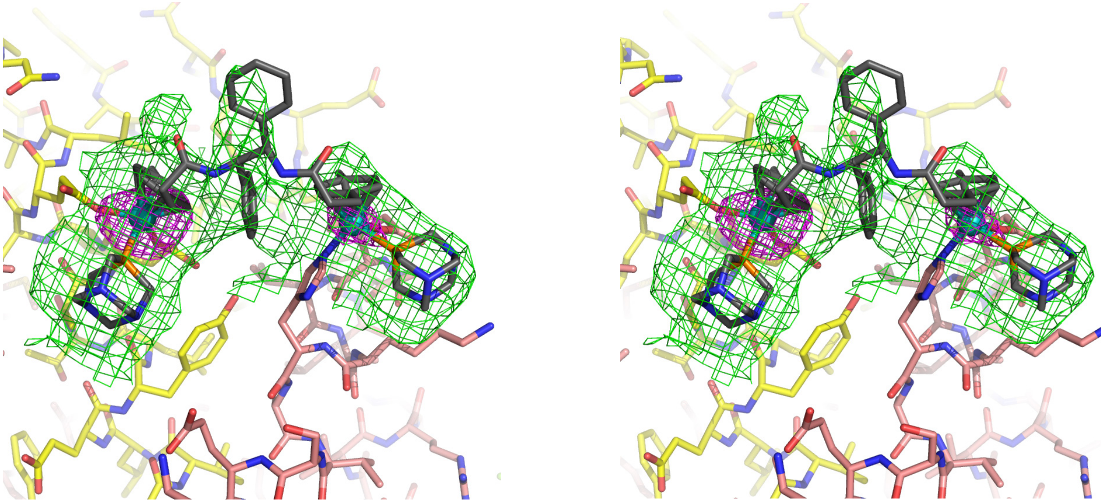

**Supplementary Figure 6.** Experimental electron density corresponding to the adduct in the X-ray crystal structure of RR-treated NCP, in stereo view. An $F_O - F_C$ omit electron density map (green; contoured at 2σ; RR atoms omitted from the model) and an anomalous difference electron density map (magenta; contoured at 4σ; indicating the locations of the ruthenium cations, teal spheres) are superimposed onto the refined model. The RU1 and RU2 sites are at the left and right, respectively. Further details can be found in Fig. 2.

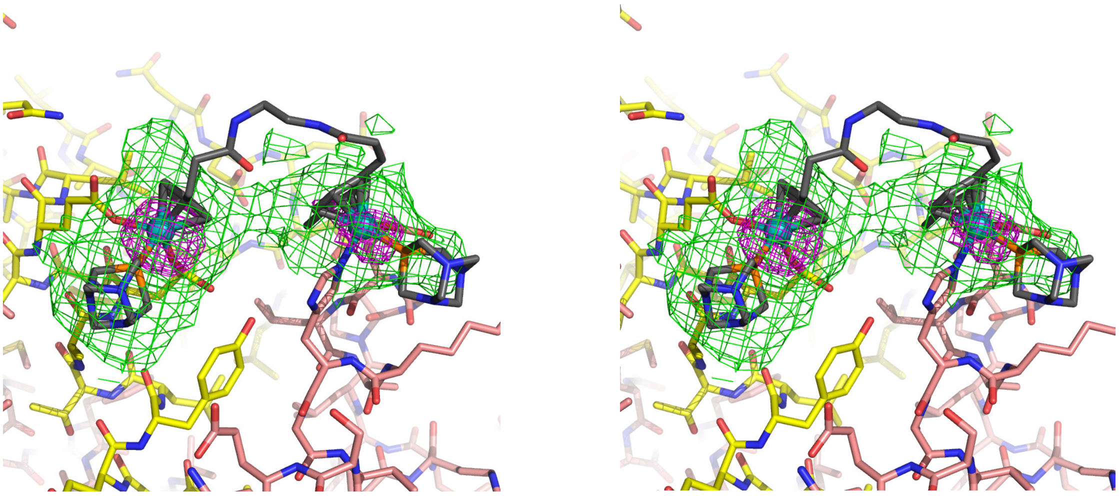

**Supplementary Figure 7.** Experimental electron density corresponding to the adduct in the X-ray crystal structure of C2-treated NCP, in stereo view. An $F_O{-}F_C$ omit electron density map (green; contoured at 2σ; C2 atoms omitted from the model) and an anomalous difference electron density map (magenta; contoured at 4σ; indicating the locations of the ruthenium cations, teal spheres) are superimposed onto the refined model. The RU1 and RU2 sites are at the left and right, respectively. Further details can be found in Fig. 2d.

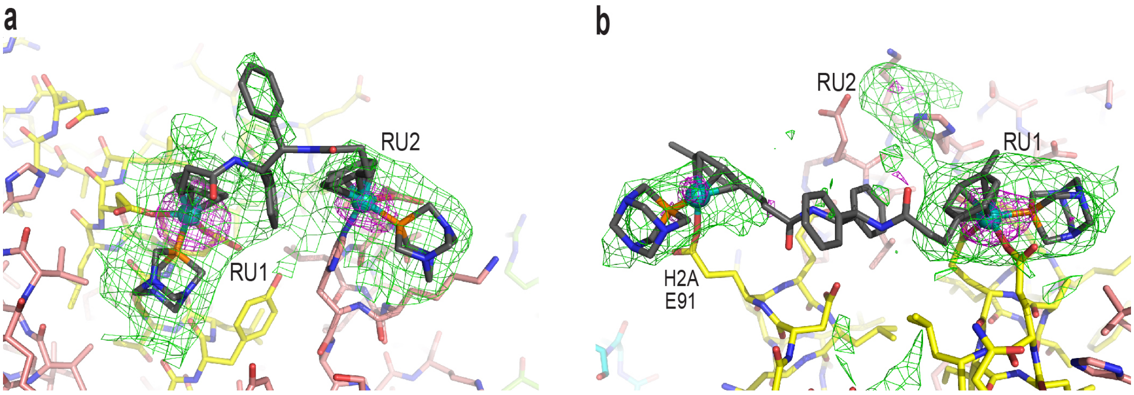


**Supplementary Figure 8.** Experimental electron density corresponding to the adducts in the X-ray crystal structures of SS-treated (**a**) and RS-treated (**b**) NCP. An $F_O - F_C$ omit electron density map (green; contoured at 2σ (a) and 3σ (b); SS/RS atoms omitted from the model) and an anomalous difference electron density map (magenta; contoured at 4σ (a) and 3σ (b); indicating the locations of the ruthenium cations, teal spheres) are superimposed onto the refined model.

**P-values for Fisher distances**

| | C10 | C2 | PEG | RAPTA | RR | Untreated |
|---|---|---|---|---|---|---|
| C10 | 1.0000 | 0.0898 | 0.0921 | **< 0.0001** | 0.5402 | 0.5615 |
| C2 | 0.0898 | 1.0000 | 0.6489 | **< 0.0001** | 0.0556 | 0.3013 |
| PEG | 0.0921 | 0.6489 | 1.0000 | **< 0.0001** | 0.0274 | 0.1792 |
| RAPTA | **< 0.0001** | **< 0.0001** | **< 0.0001** | 1.0000 | **< 0.0001** | **< 0.0001** |
| RR | 0.5402 | 0.0556 | 0.0274 | **< 0.0001** | 1.0000 | 0.7943 |
| Untreated | 0.5615 | 0.3013 | 0.1792 | **< 0.0001** | 0.7943 | 1.0000 |

**Mahalanobis distances**

| | C10 | C2 | PEG | RAPTA | RR | Untreated |
|---|---|---|---|---|---|---|
| C10 | 0.00 | 2.56 | 2.54 | 150.08 | 0.79 | 0.75 |
| C2 | 2.56 | 0.00 | 0.59 | 178.46 | 3.05 | 1.37 |
| PEG | 2.54 | 0.59 | 0.00 | 189.04 | 3.79 | 1.88 |
| RAPTA | 150.08 | 178.46 | 189.04 | 0.00 | 142.46 | 156.15 |
| RR | 0.79 | 3.05 | 3.79 | 142.46 | 0.00 | 0.37 |
| Untreated | 0.75 | 1.37 | 1.88 | 156.15 | 0.37 | 0.00 |

**Supplementary Figure 9.** Statistical analysis for the cell cycle data (Fig. 4; treatment with binuclear agent or RAPTA-C [RAPTA] and untreated control). P-values for Fisher distances (bold values signify statistically significant differences) and the Mahalanobis distances are given.

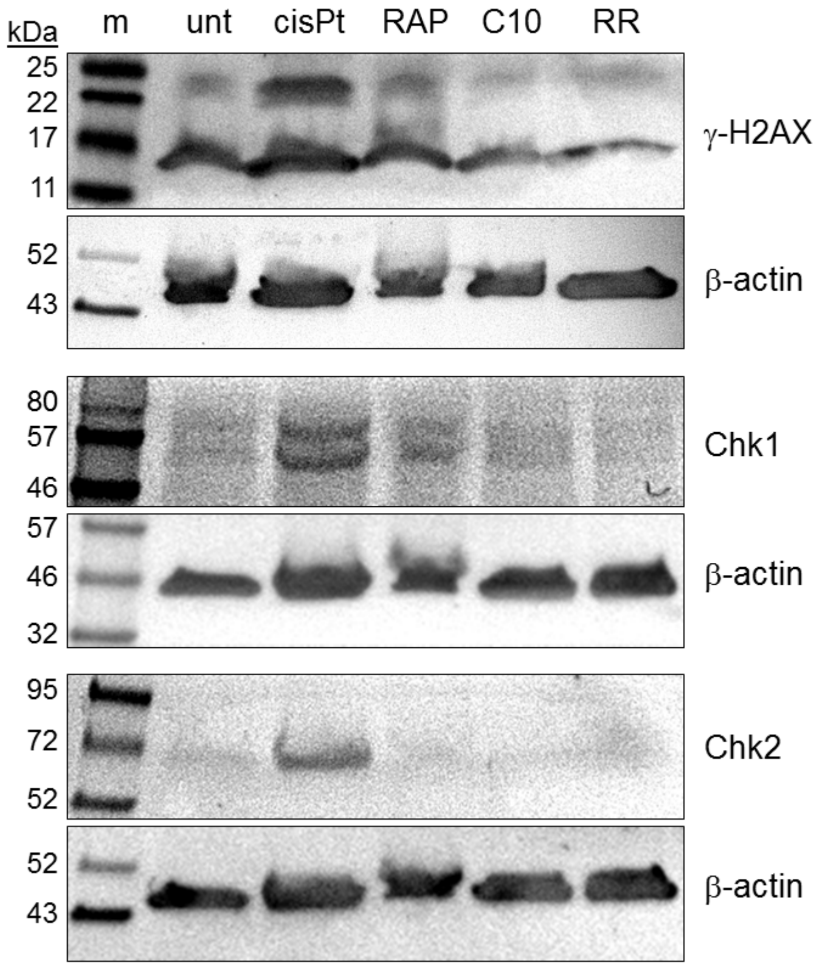


**Supplementary Figure 10.** Western blot analysis for DNA damage response markers. Cellular samples correspond to untreated (unt) or treated with either cisplatin (cisPt), RAPTA-C (RAP), C10 or RR. Molecular weight protein markers are designated by "m". An empty lane between the 'm' and 'unt' lanes was removed for the γ-H2AX blot. Full blots are shown in Supplementary Fig. 11.

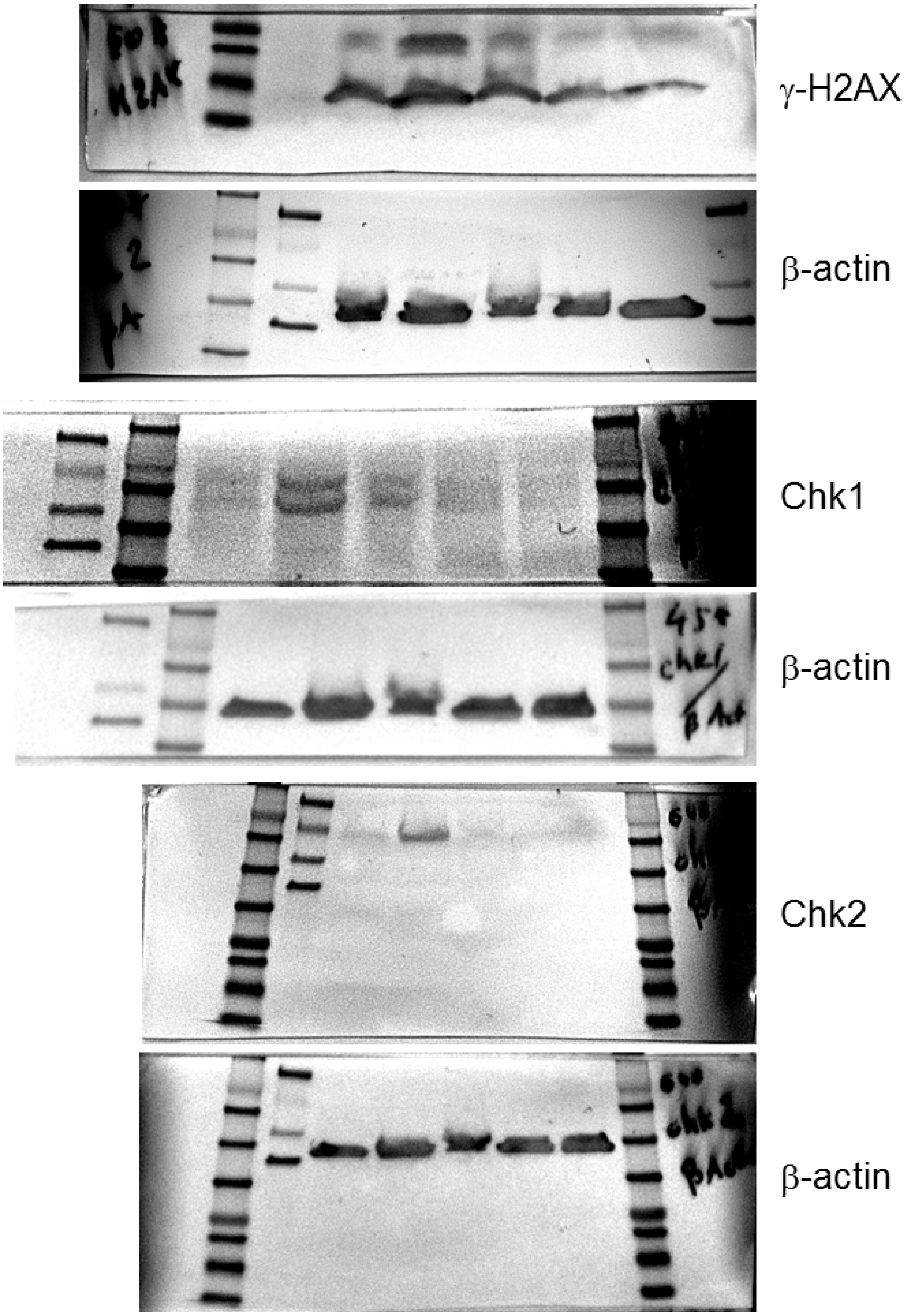


**Supplementary Figure 11.** Western blot analysis for DNA damage response markers. Uncropped scans corresponding to blots in Supplementary Fig.10.

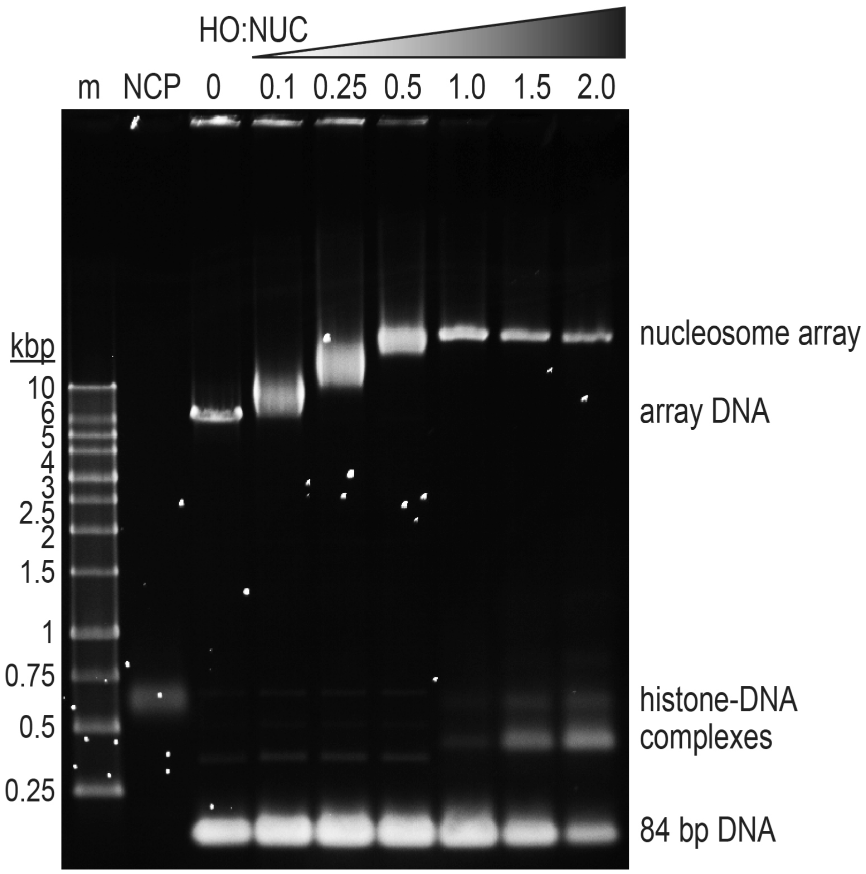


**Supplementary Figure 12.** Agarose gel analysis of nucleosome array reconstitutions. Numbers indicate the molar stoichiometry of histone octamer (HO) to nucleosome (NUC). At the left are 250–10,000 bp DNA marker (m) and nucleosome core particle (NCP) for reference. Note that saturation of the array DNA with histone octamer is achieved around a HO:NUC stoichiometry of 1.0, above which point the lower octamer-binding-affinity competitor DNA (84 bp DNA; present to prevent oversaturation of the array DNA) begins to heavily accumulate histone proteins (histone-DNA complexes).

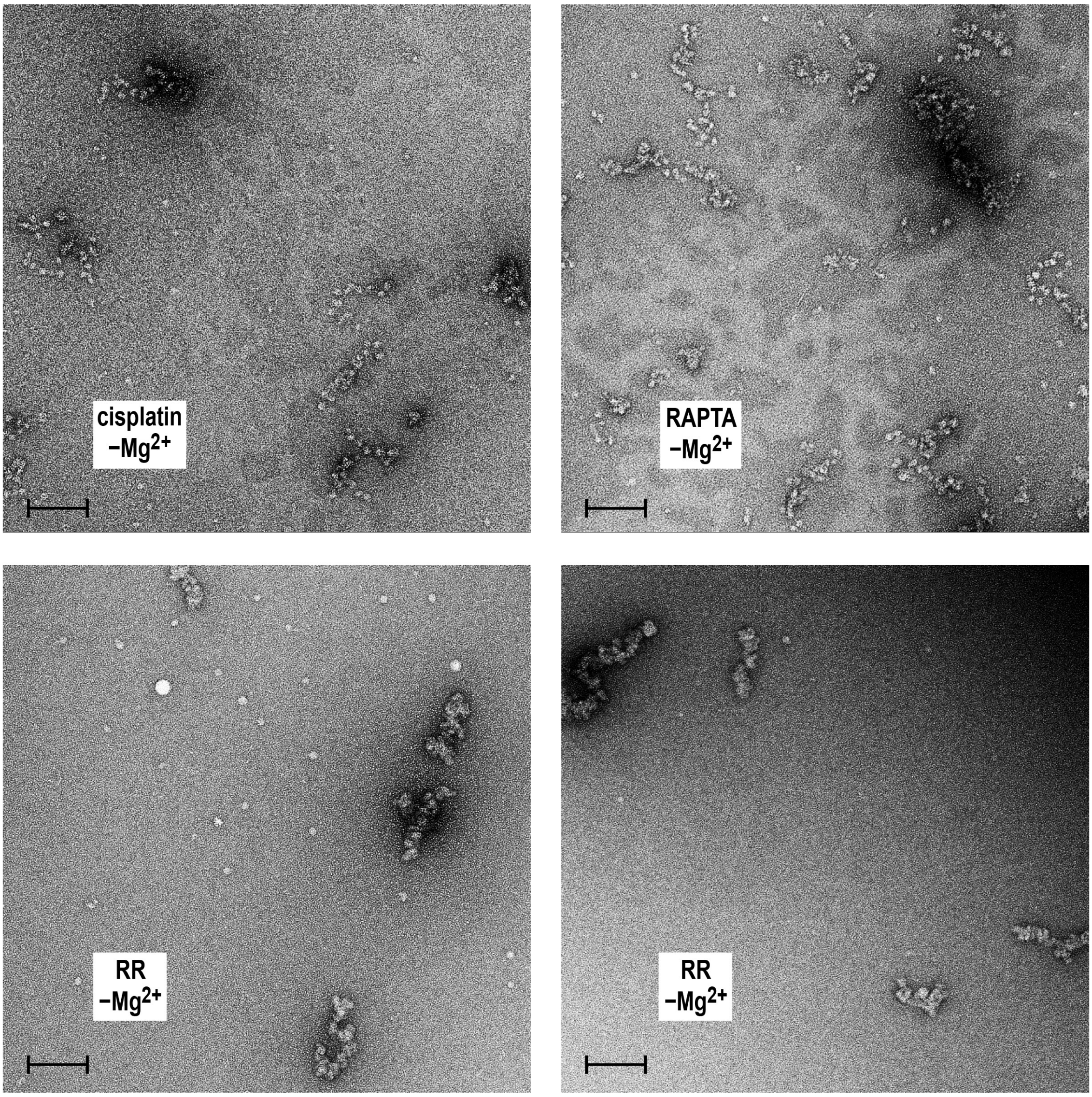


**Supplementary Figure 13.** Electron microscopy of negatively stained drug- and binuclear-treated nucleosome array. Samples are cisplatin-, RAPTA-C (RAPTA)- and RR-treated (2 images) array, all in the absence of divalent metal. The black scale bar inset corresponds to 100 nm.

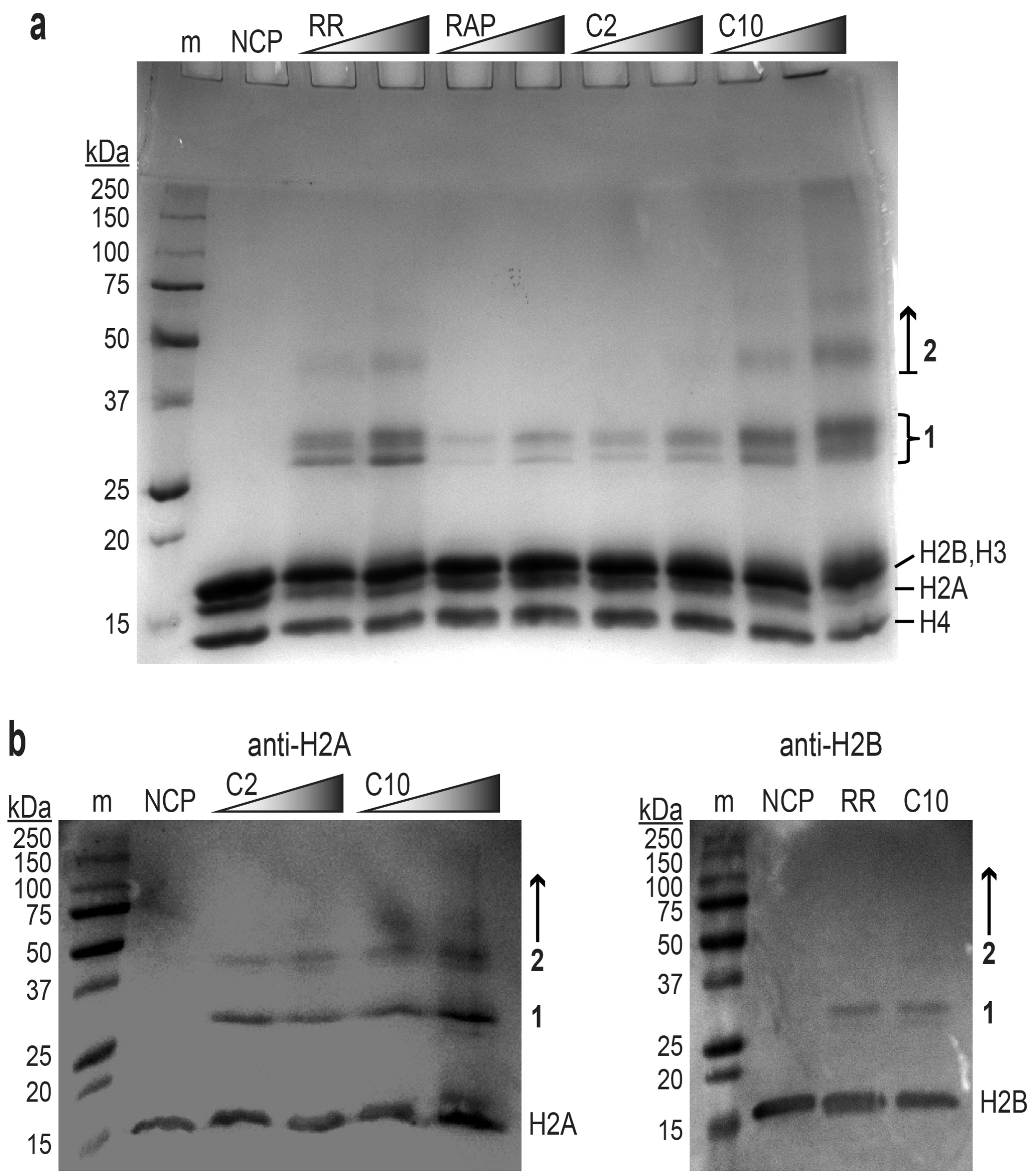


**Supplementary Figure 14.** Histone protein cross-linking analysis of NCP treated with RAPTA-C (RAP) or binuclear agent. (**a**) SDS-PAGE analysis shows multiple types of multimeric histone protein species arising from compound treatment. The cross-linked histones are categorized into two groupings (1, 2). Similar types of species in group 1 apparently occur for both RAPTA-C and binuclear treatments, although these are more prominent for the binuclears, which also display a third or additional species not seen for the mononuclear RAPTA drug. On the other hand, species in group 2 are only found to occur for the binuclear treatments. (**b**) Western blot analysis of binuclear-

treated NCP, coinciding with detection of H2A (left) or H2B (right). The analysis indicates the presence of both H2A and H2B within the group 1 species, whereas only H2A is clearly evident in group 2. Consistent with the enrichment of H2A in the cross-linked species, note the heavy depletion of the monomeric form of H2A in the binuclear-treated samples (a). (**a**,**b**) "NCP" and "m" designate untreated NCP control and molecular weight protein marker, respectively.

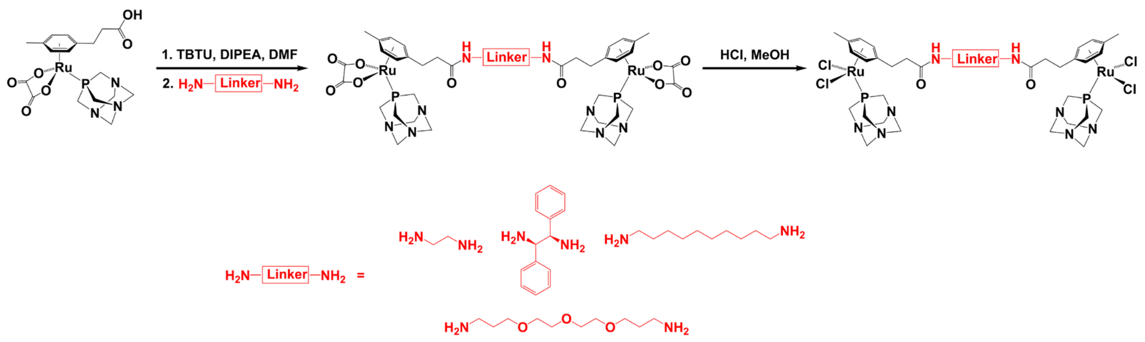


**Supplementary Figure 15.** Synthetic route utilized to produce the binuclear compounds.

**Supplementary Table 1.** Data collection and refinement statistics for NCP treated with RR, SS, RS or C2

| | **RR** | **SS** | **RS** | **C2** |
|---|---|---|---|---|
| [Binuclear] (mM) | 1 | 1 | 2 | 1 |
| Duration (h) | 19 | 43 | 43 | 16 |
| Histones | *H. sapiens* | *H. sapiens* | *H. sapiens* | *X. laevis* |
| | | | | |
| **Data collection*** | | | | |
| Space group | $P2_12_12_1$ | $P2_12_12_1$ | $P2_12_12_1$ | $P2_12_12_1$ |
| Cell dimensions | | | | |
| $a$ (Å) | 108.19 | 107.76 | 107.21 | 106.84 |
| $b$ (Å) | 109.40 | 109.56 | 109.67 | 109.73 |
| $c$ (Å) | 174.82 | 175.22 | 182.04 | 182.53 |
| Resolution (Å) | 2.60–58.3 (2.60–2.74) | 2.87–70.4 (2.87–3.03) | 2.82–70.7 (2.82–2.97) | 2.63–48.8 (2.63–2.77) |
| $R_{merge}$ (%) | 5.0 (50.5) | 5.5 (49.8) | 6.2 (50.2) | 2.8 (47.1) |
| $I / \sigma I$ | 15.0 (2.5) | 15.6 (2.9) | 13.3 (2.4) | 31.8 (1.8) |
| Completeness (%) | 99.9 (99.6) | 99.8 (99.1) | 99.9 (99.6) | 94.5 (71.6) |
| Redundancy | 5.8 (5.4) | 5.7 (5.3) | 5.8 (5.1) | 5.3 (2.4) |
| | | | | |
| **Refinement** | | | | |
| Resolution (Å) | 2.60–58.3 | 2.87–70.4 | 2.82–70.7 | 2.63–47.2 |
| No. reflections | 63,050 | 47,071 | 51,315 | 57,584 |
| $R_{work}$ / $R_{free}$ (%) | 23.5 / 27.0 | 22.7 / 26.6 | 22.8 / 26.4 | 20.8 / 25.4 |
| No. atoms | 12,099 | 12,099 | 12,099 | 12,111 |
| Protein | 6,094 | 6,094 | 6,094 | 6,086 |
| DNA | 5,939 | 5,939 | 5,939 | 5,939 |
| Solvent | 6 | 6 | 6 | 38 |
| Adduct | 60 | 60 | 60 | 48 |
| $B$-factors (Å$^2$) | 111 | 117 | 103 | 95 |
| Protein | 80 | 84 | 74 | 67 |
| DNA | 142 | 150 | 133 | 124 |
| Solvent | 147 | 153 | 127 | 75 |
| Adduct | 161 | 173 | 192 | 175 |
| R.m.s. deviations | | | | |
| Bond lengths (Å) | 0.009 | 0.008 | 0.006 | 0.009 |
| Bond angles (°) | 1.45 | 1.41 | 1.20 | 1.45 |

Single crystal data.
Values in parentheses are for the highest-resolution shell.

**Supplementary Table 2.** Data collection statistics for NCP treated with PEG, C10, PEGOx or C10Ox (chloride ligands substituted with oxalate)

| | **PEG** | **C10** | **PEGOx** | **C10Ox** |
|---|---|---|---|---|
| [Binuclear] (mM) | 1 | 2 | 2 | 2 |
| Duration (h) | 44 | 39 | 66 | 66 |
| Histones | *X. laevis* | *X. laevis* | *X. laevis* | *X. laevis* |
| | | | | |
| **Data collection*** | | | | |
| Space group | $P2_12_12_1$ | $P2_12_12_1$ | $P2_12_12_1$ | $P2_12_12_1$ |
| Cell dimensions | | | | |
| *a* (Å) | 107.10 | 107.40 | 106.79 | 106.90 |
| *b* (Å) | 109.89 | 110.15 | 109.79 | 109.90 |
| *c* (Å) | 182.62 | 181.51 | 182.40 | 182.71 |
| Resolution (Å) | 2.83–48.9 (2.83–2.99) | 3.70–94.1 (3.70–3.90) | 2.60–48.8 (2.60–2.74) | 2.60–48.9 (2.60–2.74) |
| $R_{merge}$ (%) | 2.6 (48.8) | 4.2 (49.7) | 3.5 (46.6) | 2.6 (35.7) |
| $I / \sigma I$ | 30.6 (1.4) | 13.4 (2.0) | 24.2 (1.5) | 37.6 (3.0) |
| Completeness (%) | 98.0 (88.7) | 99.7 (98.3) | 97.9 (89.0) | 89.1 (51.4) |
| Redundancy | 5.0 (2.0) | 5.2 (3.2) | 5.1 (2.2) | 5.6 (3.7) |

Single crystal data. Values in parentheses are for the highest-resolution shell.

**Supplementary Table 3.** Data collection statistics for NCP treated with C2Ox, SSOx or RSOx (chloride ligands substituted with oxalate)

| | **C2Ox** | **SSOx** | **RSOx** |
|---|---|---|---|
| [Binuclear] (mM) | 2 | 1 | 2 |
| Duration (h) | 66 | 23 | 67 |
| Histones | *X. laevis* | *X. laevis* | *X. laevis* |
| | | | |
| **Data collection*** | | | |
| Space group | $P2_12_12_1$ | $P2_12_12_1$ | $P2_12_12_1$ |
| Cell dimensions | | | |
| *a* (Å) | 106.94 | 106.80 | 106.89 |
| *b* (Å) | 109.82 | 109.81 | 109.81 |
| *c* (Å) | 182.73 | 182.51 | 182.59 |
| Resolution (Å) | 2.60–48.9 (2.60–2.74) | 2.59–48.8 (2.59–2.73) | 2.60–48.8 (2.60–2.74) |
| $R_{merge}$ (%) | 2.8 (43.8) | 3.6 (48.5) | 3.0 (30.3) |
| $I / \sigma I$ | 35.3 (2.4) | 26.4 (1.9) | 29.2 (1.8) |
| Completeness (%) | 96.6 (83.2) | 97.7 (85.0) | 95.5 (76.4) |
| Redundancy | 5.7 (3.6) | 5.5 (3.0) | 5.1 (1.9) |

Single crystal data. Values in parentheses are for the highest-resolution shell.

**Supplementary Table 4.** Binuclear binding sites in crystals of the NCP

| Agent Treatment (mM, h) | | | Resolution (Å) | Anomalous Peak (σ) | | |
|---|---|---|---|---|---|---|
| Agent | Conc. | Duration | | RU1 | RU2 | H2A E91 |
| C2 | 1 | 16 | 2.65 | 8.5 | 6.2 | |
| | 1 | 38 | 2.88 | 7.9 | 8.3 | |
| | 2 | 16 | 2.83 | 6.9 | 6.9 | |
| | 2 | 38 | 3.47 | 7.4 | 7.0 | |
| C10 | 2 | 16 | 2.77 | 4.6 | | |
| | 2 | 39 | 3.70 | 6.7 | 3.2 | |
| PEG | 1 | 22 | 2.75 | 6.6 | 3.9 | |
| | 1 | 44 | 2.83 | 9.3 | 5.7 | |
| | 1 | 67 | 3.55 | 6.3 | 6.6 | |
| | 2 | 15 | 2.81 | 5.7 | | |
| | 2 | 39 | 3.30 | 8.4 | 5.9 | |
| RR | 1 | 19 | 2.60 | 11.5 | 5.9 | |
| | 1 | 42 | 3.15 | 6.4 | 3.0 | |
| SS | 1 | 19 | 2.50 | 3.5 | 3.4 | |
| | 1 | 43 | 2.87 | 8.9 | 6.4 | |
| RS | 1 | 19 | 2.28 | 4.9 | | |
| | 1 | 43 | 2.82 | 6.8 | 3.4 | 3.7 |
| | 2 | 17 | 2.45 | 9.8 | | |
| C2Ox | 1 | 22 | 2.00 | | | |
| | 1 | 44 | 2.60 | | | |
| | 2 | 40 | 2.65 | | | |
| | 2 | 66 | 2.60 | | | |
| C10Ox | 2 | 66 | 2.60 | | | |
| PEGOx | 1 | 22 | 2.60 | | | |
| | 2 | 66 | 2.60 | | | |
| SSOx | 1 | 23 | 2.55 | | | |
| | 1 | 44 | 3.00 | | | |
| | 2 | 66 | 2.65 | | | |
| RSOx | 2 | 41 | 2.60 | | | |
| | 2 | 67 | 2.65 | | | |

Peak heights from anomalous difference electron density maps ( $\geq 3\sigma$)
