# Supplementary material for: Nucleosome acidic patch-targeting binuclear ruthenium compounds induce aberrant chromatin condensation
Source: Nat Commun. 2017 Nov 17;8:1575. doi: 10.1038/s41467-017-01680-4 (PMC5691193; doi:10.1038/s41467-017-01680-4)
Supplement: Supplementary file 3 — Description of Additional Supplementary Files [file 41467_2017_1680_MOESM3_ESM.pdf]

Description of Additional Supplementary Files

File Name: Supplementary Movie 1
Description: Control, untreated cells. Live fluorescence imaging of drug- and binuclear-treated cells over the course of 24 h. Nuclear chromatin is visible by virtue of the incorporated H2B-EGFP histone fusion protein. Frames have been selected to show cell division (untreated) or a cellular crisis event (aberrant chromatin condensation/apoptosis).

File Name: Supplementary Movie 2
Description: Cisplatin-treated. Live fluorescence imaging of drug- and binuclear-treated cells over the course of 24 h. Nuclear chromatin is visible by virtue of the incorporated H2B-EGFP histone fusion protein. Frames have been selected to show cell division (untreated) or a cellular crisis event (aberrant chromatin condensation/apoptosis).

File Name: Supplementary Movie 3
Description: RAPTA-C-treated. Live fluorescence imaging of drug- and binuclear-treated cells over the course of 24 h. Nuclear chromatin is visible by virtue of the incorporated H2B-EGFP histone fusion protein. Frames have been selected to show cell division (untreated) or a cellular crisis event (aberrant chromatin condensation/apoptosis).

File Name: Supplementary Movie 4
Description: PEG-treated. Live fluorescence imaging of drug- and binuclear-treated cells over the course of 24 h. Nuclear chromatin is visible by virtue of the incorporated H2B-EGFP histone fusion protein. Frames have been selected to show cell division (untreated) or a cellular crisis event (aberrant chromatin condensation/apoptosis).

File Name: Supplementary Movie 5
Description: RR-treated. Live fluorescence imaging of drug- and binuclear-treated cells over the course of 24 h. Nuclear chromatin is visible by virtue of the incorporated H2B-EGFP histone fusion protein. Frames have been selected to show cell division (untreated) or a cellular crisis event (aberrant chromatin condensation/apoptosis).

File Name: Supplementary Movie 6
Description: C2-treated. Live fluorescence imaging of drug- and binuclear-treated cells over the course of 24 h. Nuclear chromatin is visible by virtue of the incorporated H2B-EGFP histone fusion protein. Frames have been selected to show cell division (untreated) or a cellular crisis event (aberrant chromatin condensation/apoptosis).

File Name: Supplementary Movie 7
Description: C10-treated. Live fluorescence imaging of drug- and binuclear-treated cells over the course of 24 h. Nuclear chromatin is visible by virtue of the incorporated H2B-EGFP histone fusion protein. Frames have been selected to show cell division (untreated) or a cellular crisis event (aberrant chromatin condensation/apoptosis).
